# A polyalanine peptide derived from polar fish with anti-infectious activities

**DOI:** 10.1038/srep21385

**Published:** 2016-02-26

**Authors:** Marlon H. Cardoso, Suzana M. Ribeiro, Diego O. Nolasco, César de la Fuente-Núñez, Mário R. Felício, Sónia Gonçalves, Carolina O. Matos, Luciano M. Liao, Nuno C. Santos, Robert E. W. Hancock, Octávio L. Franco, Ludovico Migliolo

**Affiliations:** 1 Programa de Pós-Graduação em Ciências Genômicas e Biotecnologia, Universidade Católica de Brasília, Brasília-DF, Brazil; 2 Centro de Análises Proteômicas e Bioquímicas, Pós-Graduação em Ciências Genômicas e Biotecnologia, Universidade Católica de Brasília, Brasília-DF, Brazil; 3 S-inova, Programa de Pós-Graduação em Biotecnologia, Universidade Católica Dom Bosco, Campo Grande-MS, Brazil; 4 Programa de Pós-Graduação em Patologia Molecular, Faculdade de Medicina, Universidade de Brasília, Brasília-DF, Brazil; 5 Instituto de Química, Universidade Federal de Goiás, Goiânia-GO, Brazil; 6 Research Laboratory of Electronics, Massachusetts Institute of Technology (MIT), Cambridge, Massachusetts, USA; 7 Centre for Microbial Diseases and Immunity Research, Department of Microbiology and Immunology, University of British Columbia, Vancouver, Canada; 8 Instituto de Medicina Molecular, Faculdade de Medicina, Universidade de Lisboa, Lisbon, Portugal; 9 Synthetic Biology Group, MIT Synthetic Biology Center, Research Laboratory of Electronics, Department of Biological Engineering, Department of Electrical Engineering and Computer Science, Massachusetts Institute of Technology, Cambridge, Massachusetts, United States of America. Broad Institute of MIT and Harvard

## Abstract

Due to the growing concern about antibiotic-resistant microbial infections, increasing support has been given to new drug discovery programs. A promising alternative to counter bacterial infections includes the antimicrobial peptides (AMPs), which have emerged as model molecules for rational design strategies. Here we focused on the study of *Pa*-MAP 1.9, a rationally designed AMP derived from the polar fish *Pleuronectes americanus*. *Pa*-MAP 1.9 was active against Gram-negative planktonic bacteria and biofilms, without being cytotoxic to mammalian cells. By using AFM, leakage assays, CD spectroscopy and *in silico* tools, we found that *Pa*-MAP 1.9 may be acting both on intracellular targets and on the bacterial surface, also being more efficient at interacting with anionic LUVs mimicking Gram-negative bacterial surface, where this peptide adopts α-helical conformations, than cholesterol-enriched LUVs mimicking mammalian cells. Thus, as bacteria present varied physiological features that favor antibiotic-resistance, *Pa*-MAP 1.9 could be a promising candidate in the development of tools against infections caused by pathogenic bacteria.

In recent decades, improvements in the prevention and treatment of infectious diseases caused by pathogenic microorganisms has been of great importance in reducing morbidity and mortality, leading to a better quality of life and longer life expectancy^1^. However, antibiotics have been widely and sometimes indiscriminately used, which has resulted in the emergence of pathogens with multi-drug resistance in a wide range of bacterial species, including *Escherichia coli*, *Staphylococcus aureus, Pseudomonas aeruginosa, Klebsiella pneumoniae* and many other species^2^. Conversely, the rate of discovery of new antibiotics has steadily plummeted. Additionally, the occurrence and treatment of biofilm infections has appeared as one of the biggest challenges in the medical field with no available antibiotics that were developed to treat such infections. Biofilms are characterized as being a structured consortium of microorganisms connected by a complex matrix composed of polysaccharide(s), protein and DNA, that grows on biotic or abiotic surfaces via a multistage process^3^. It has been established that pathogenic bacteria are predominantly organized in biofilms, which are the cause of 65% to 80% of all bacterial infections in humans^3^,^4^. Biofilm growth represents a unique growth state whereby bacteria have major physiological and organizational differences, in particular leading to 10- to 1000- fold increased (adaptive) resistant to conventional antibiotics^3^,^4^.

In this context, investigating and improving on natural compounds has been of great interest in the search for promising alternatives to conventional medicines. In recent years, cationic amphipathic peptides termed anti-microbial peptides (AMPs) have been widely investigated as a promising alternative for the treatment of infections caused by pathogenic microorganisms^5^. These molecules have been isolated from a wide number of organisms including plants^6^, animals^7^ and bacteria^8^. These peptides tend to have multiple targets that can include the cytoplasmic membrane permeability barrier, macromolecular processes dependent on the membrane, including cell wall biosynthesis and cell division, and/or other intracellular targets including RNA and protein synthesis^5^. Moreover, recent evidence indicates that AMPs’ ability to act on intracellular targets can either occur as a major mechanism of action after having crossed the membrane without causing disruptive processes, or as a secondary and/or additional mechanism to membrane disruption^9^.

Currently, some of the largest challenges in working with AMPs involve their cytotoxicity against mammalian cells, as well as their cost of production. Thus, the rational design of AMPs has gained great prominence in the scientific field, aiming to develop AMPs that are more active, less cytotoxic and possible to produce on an industrial scale^10^. Early studies of rational design played an important role in the identification of optimal AMP physicochemical properties, such as appropriate hydrophobicity, charge and amphipathic structural arrangement, which are all directly related with their antimicrobial activities. Template-based designs taking in account charge and amphipathicity have also been used based on modified amino acid residues of well-known AMPs in order to improve their activities^5^. Jiang and co-workers^11^ have recently introduced a new concept of template-based design involving the arrangement of lysine and arginine residues in the center of the non-polar region of amphipathic α-helical AMPs in order to enhance peptide’s selectivity against both eukaryotic and prokaryotic cell membranes. In addition to these strategies, biophysical studies have been used to evaluate AMP activities and design improved analogues by predicting and characterizing their structures in different environments, as well as performing molecular modelling, dynamics and docking simulations at atomic levels^5^.

This work focuses on a novel polyalanine-rich cationic AMP, named *Pa*-MAP 1.9, that was rationally designed based on a synthetic multifunctional peptide (*Pa*-MAP) derived from HPLC-8, a peptide originally isolated from the polar fish *Pleuronectes americanus*^12^. Here, we report the antimicrobial activities of this peptide, mainly against *Enterococcus faecalis*, *S.aureus*, *E. coli* and *K. pneumoniae* planktonic bacteria, as well as *K. pneumoniae* and *E. coli* biofilms. Furthermore, biophysical experiments, using circular dichroism (CD), fluorescence spectroscopy and atomic force microscopy (AFM), in combination with *in silico* studies such as molecular modelling, dynamics and docking, were performed to obtain insights into the structure of *Pa*-MAP 1.9, as well as its mechanism of action.

## Results

### *Pa*-MAP 1.9 synthesis and mass spectrometry analysis

*Pa*-MAP 1.9 (NH_2_-LAAKLTKAATKLTAALTKLAAALT-COOH) was designed, and synthetized by Fmoc strategy loosely based on the sequence of a previously described multifunctional peptide, *Pa*-MAP (NH_2_-HTASDAAAAAALTAANAAAAAAASMA-COOH)^12^, in which the net charge and hydrophobic moment were increased, hydrophobic amino acid frequency was decreased (since high hydrophobicity favors toxicity) and alanine residues were distributed along the molecule in order to obtain a linear cationic peptide with predicted helical stretches. MALDI-ToF analysis showed greater than 95% purity and an ion mass of 2668.0 m/z, in agreement with the theoretical calculated molecular mass for this peptide (Fig. S1).

### *In vitro* antimicrobial assays

**Figure 1:**
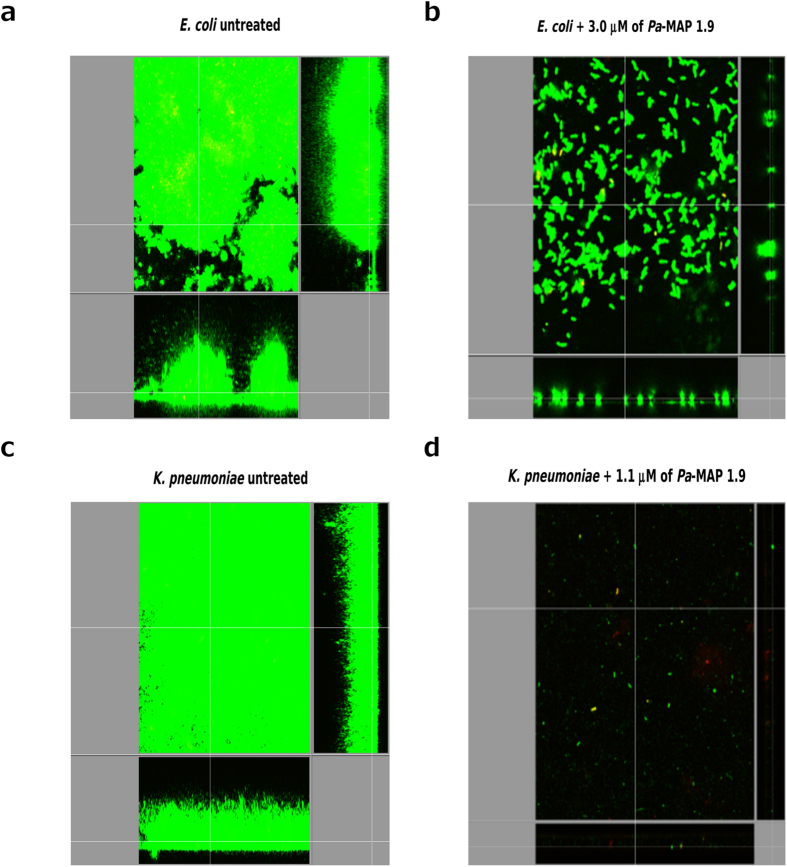
Flow-cell analysis of *E. coli* and *K. pneumoniae* biofilm formation in the absence and presence of *Pa*-MAP 1.9. Pre-formed *E. coli* biofilm before (**a**) and after (**b**) treatment with 3.0 μM of *Pa*-MAP 1.9 (**b**). Pre-formed *K. pneumoniae* biofilm before (**c**) and after (**d**) treatment with 1.1 μM of *Pa*-MAP 1.9.

*Pa*-MAP 1.9 was evaluated for its ability to inhibit the growth of different bacteria grown planktonically and in biofilms. *Pa*-MAP 1.9 was able to inhibit the growth of *Enterococcus faecalis*, *E. coli* and *K. pneumoniae* planktonic cells with minimal inhibitory concentrations (MICs) of 1.5, 6–12 and 24–96 μM, respectively (Table 1). However, no antibacterial activity was observed against *P. aeruginosa* and *S. aureus*, even at the maximum concentration used for this assay (115 μM). Against bacteria in their biofilm growth state, *Pa*-MAP 1.9 was considerably more potent, with (MBICs) of 3.0 and 1.1 μM (Table 1) against *E. coli* and *K. pneumoniae* biofilms respectively. At these same concentrations, flow cell analysis revealed that pre-formed *E. coli* and *K. pneumoniae* biofilms (Fig. 1a,c) were strongly or completely inhibited, with a strong decrease in biofilm volume and height (Fig. 1b,d).

### Hemolytic and cytotoxicity assays

*Pa*-MAP 1.9 did not show neither hemolytic activity against human erythrocytes or cytotoxic effects against RAW 264.7 monocyte cell line up to 115 μM, the maximum concentration used in the bioassays (Table 1).

### Atomic force microscopy analysis

**Figure 2:**
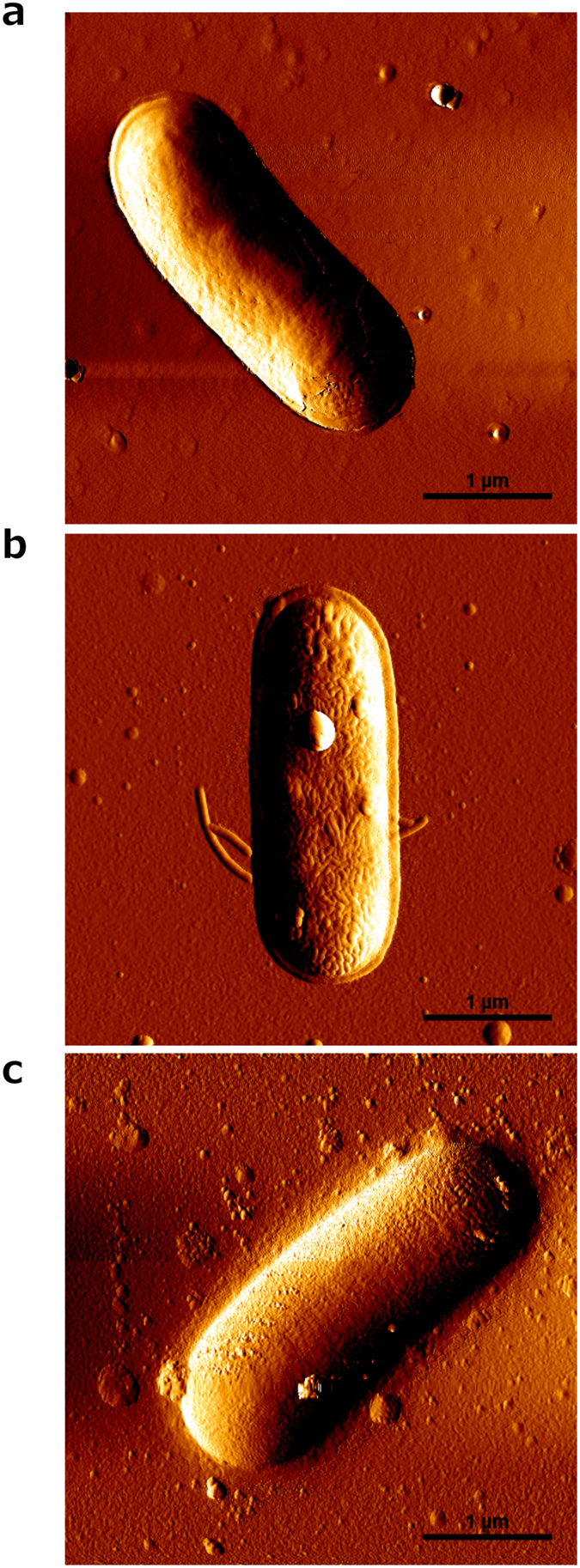
Atomic force microscopy (AFM) images of *E. coli* in the absence and presence of *Pa*-MAP 1.9. Untreated bacteria (control) (**a**), bacteria treated with 6 μM (**b**) and 300 μM (**c**) of *Pa*-MAP 1.9. Total scanning area per image: 4 × 4 μm^2^; scale bar: 1 μM.

AFM was used to image the possible AMP-induced damage of the Gram-negative bacterial envelope. At 6 μM (MIC), *Pa*-MAP 1.9 induced slight morphological changes in the form of increased surface roughness in *E. coli* (Fig. 2b), relative to the untreated control (Fig. 2a). Damage was more evident at 300 μM (50-fold the MIC), where substantial bacterial surface disruption was observed (Fig. 2c).

### Permeabilization of lipid vesicles

**Figure 3:**
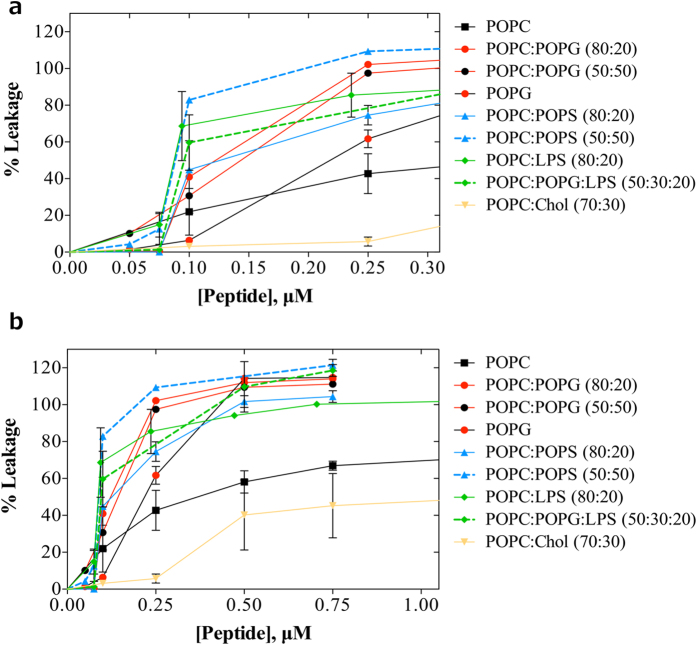
Leakage experiments performed with increasing concentrations of *Pa*-MAP 1.9 against large unilamellar vesicles. Percentage of carboxyfluorescein release (CF) in unilamellar vesicles composed of different proportions of POPC, POPG, POPS, LPS and cholesterol induced by different peptide concentration, ranging from 0.0 to 0.3 μM (**a**) and from 0.0 to 1 μM (**b**).

The ability of *Pa*-MAP 1.9 to disrupt large unilamellar vesicles (LUVs) with compositions roughly mimicking bacterial membranes was also analyzed. Vesicles were prepared with encapsulated carboxyfluorescein (CF), and then incubated with *Pa*-MAP 1.9 concentrations up to 1.0 μM. The percentages of leakage were evaluated as previously described^13^, by assessing the differences over time in the emission of CF (which fluoresces more strongly upon leakage from the vesicles), with 100% of leakage corresponding to full disruption of lipid vesicles. The data showed that the peptide induced leakage of vesicles containing anionic lipids such as 1-palmitoyl-2-oleoyl-sn-glycero-3-phospho-(1′–sn-glycerol) (POPG), 1-palmitoyl-2-oleoyl-sn-glycero-3-phospho-L-serine (POPS) or lipopolysaccharide (LPS), requiring only 0.25 μM peptide for almost complete permeabilization (Fig. 3a). Moreover, at 0.75 μM of *Pa*-MAP 1.9, full leakage was reached for all other vesicles containing anionic phospholipids (Fig. 3b). In contrast, zwitterionic vesicles of 1-palmitoyl-2-oleoyl-sn-glycero-3-phosphocholine (POPC) or POPC/Cholesterol (Chol) (70:30) clearly demonstrated reduced leakage, even at the maximum concentration used for this experiment (Fig. 3a,b). The results obtained demonstrate the specificity of the AMP for negatively charged membranes, such as those from Gram-negative bacteria and are consistent with the lack of toxicity for mammalian cells, which contain largely neutral or zwitterionic lipids on their external leaflet.

### Structural analysis

**Figure 4:**
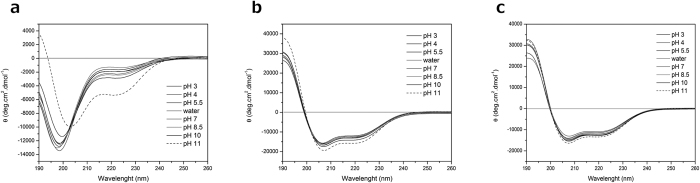
Circular dichroism analysis of *Pa*-MAP 1.9. CD spectra of *Pa*-MAP 1.9 solubilized in water (pH 3–11) (**a**) TFE 50% (v:v; pH 3–11) (**b**) and SDS 28 mM (pH 3–11) (**c**) Higher helical contents (ellipticity) were obtained/calculated at pH 11, highlighted as dashed lines in all conditions.

Circular dichroism spectroscopy studies were performed in water, 2,2,2-trifluoroethanol (TFE) 50% (v:v) and sodium dodecyl sulfate (SDS) 28 mM. When analyzed in water, the peptide showed no stable at pH values ranging from 3 to 10 (Fig. 4a). However, at pH 11 this *Pa*-MAP 1.9 produced a CD spectrum characteristic of an α-helix conformation, with two negative bands at ~205 and ~222 nm, presenting 14% of ellipticity (Fig. 4a). When analyzed in 50% TFE, *Pa*-MAP 1.9 adopted a well-defined α-helix structure at all pH values presenting a positive band at 190 nm and two negative bands at ~205 and ~222 nm (Fig. 4b). Similar results were observer for the CD spectrum in anionic lipid-like environment, where the same positive and negative bands could be observed, indicating helical conformation (Fig. 4c).

**Figure 5:**
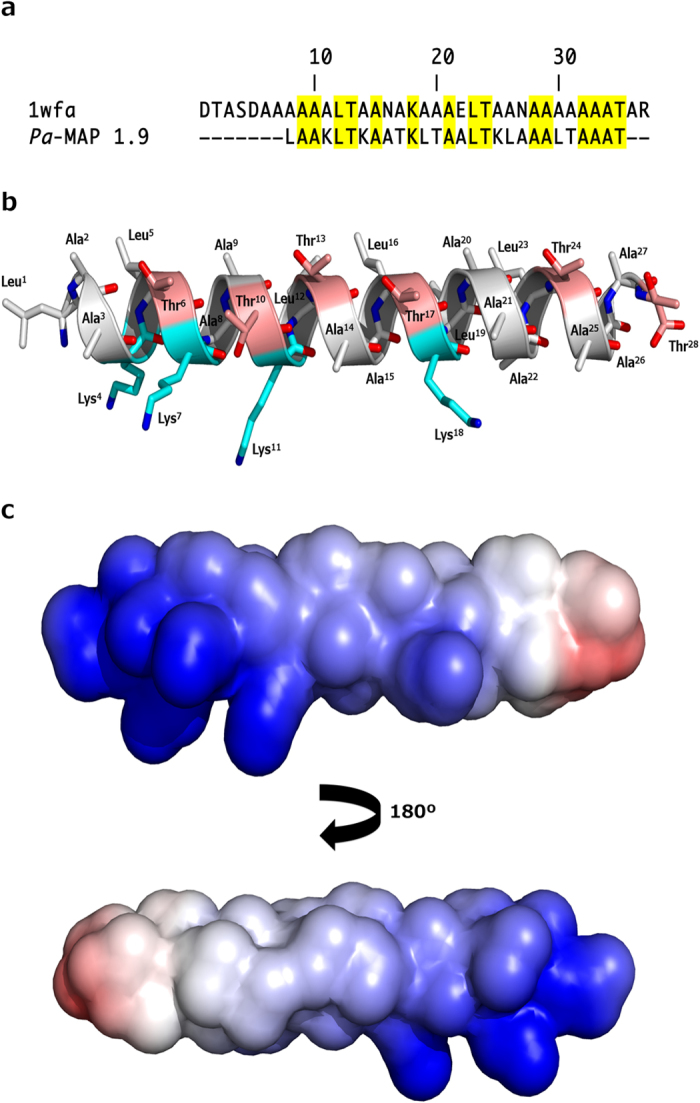
BLASTp analysis, predicted secondary structure and electrostatic potential of *Pa*-MAP 1.9. Alignment between the query (*Pa*-MAP 1.9) and template (PDB: 1wfa) primary sequences, highlighting (yellow) the identical residues (**a**). Lowest free-energy three-dimensional theoretical model for *Pa*-MAP 1.9: in white, non-polar residues; in pink, polar residues; in cyan, basic residues (**b**). Adaptive Poisson-Boltzmann solver (APBS) electrostatic potential of *Pa*-MAP 1.9; potential ranges from −10.9 kT/e (red) to + 10.1 kT/e (blue) (**c**).

To obtain insights into the three-dimensional structure of *Pa*-MAP 1.9, molecular modelling simulations were performed. The lowest free-energy theoretical model for *Pa*-MAP 1.9 revealed a well-defined α-helical conformation (Fig. 5b), and also revealed an amphipathic character (Fig. 5c) when modelled based on the antifreeze peptide (PDB code: 1wfa) isolated from *P. americanus*, which presented 58% identity over part of its sequence with the *Pa*-MAP 1.9 primary sequence (Fig. 5a). When evaluated using PROCHECK^14^, the average score for dihedral angles (ϕ-ψ, χ and ω), jointly with the main-chain covalent forces for the best model, was 0.23, which is within the expected range for a reliable structure. Moreover, a Ramachandran plot showed that 100% of the amino acid residues of *Pa*-MAP 1.9 were located in the most favorable regions. Furthermore, three-dimensional structural superposition (3DSS) analysis revealed that the root mean square deviation (RMSD) between the theoretical and experimental models was equal to 0.567 Å. Based on those data, this model was selected for further *in silico* studies.

**Figure 6:**
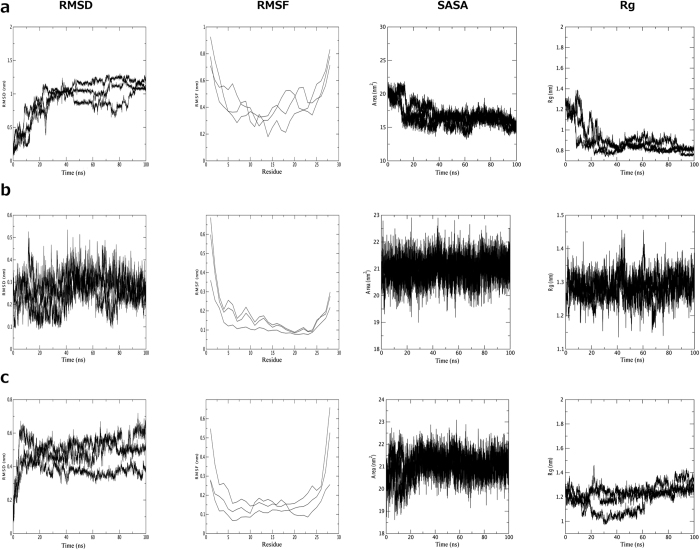
Graphical representation of physicochemical parameters resulted from molecular dynamics simulations. *Pa*-MAP 1.9 molecular dynamics simulations in water (**a**), TFE 50% (v:v) (**b**) and SDS micelle (**c**), yielding the parameters root mean square deviation (RMSD), root mean square fluctuation (RMSF), solvent-surface accessible area (SASA) and radius of gyration (Rg) for each conditions.

**Figure 7:**
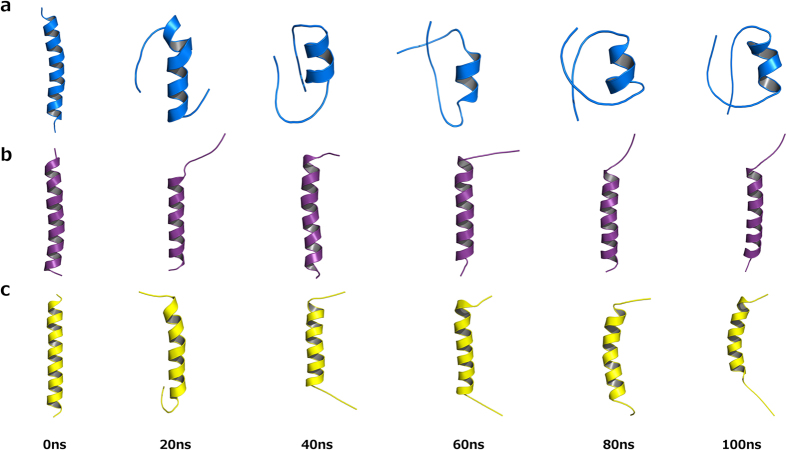
Three-dimensional theoretical structures snapshots of *Pa*-MAP 1.9 during 100 ns of molecular dynamics simulation. Evaluations were performed in water (**a**), TFE 50% (v:v) (**b**) and SDS micelle (**c**). The N-terminal region of the peptide is always at the bottom (top).

Like the CD analysis, molecular dynamics simulations of the peptide were implemented in three different environments including pure water, water and TFE mixture 50% (v:v) and water where the peptide was in contact with an SDS micelle. The simulations were performed to better understand the behavior of the three-dimensional theoretical structure of the above-described *Pa*-MAP 1.9 in different environments. After 100 ns of molecular dynamics simulations in water, it was possible to observe high values of RMSD and root mean square fluctuation (RMSF), revealing the instability of *Pa*-MAP 1.9 in this environment. In addition, it was observed a decrease in the radius of gyration (approximately from 1.3 to 0.6 nm), as well as in the solvent-accessible surface area (SASA) (Fig. 6a). These parameters, allied to the three-dimensional structures observed throughout the simulation, revealed that, consistent with the CD spectra, *Pa*-MAP 1.9 seems to lose its α-helical structure in water, also tending to hide its hydrophobic amino acid residues by adopting a coil conformation with a short central α-helix since the 20 ns of simulation (Fig. 7a). It can also be associated with a decrease of approximately 0.8 nm^2^ in the SASA (Fig. 6a). On the other hand, simulations performed in water and TFE demonstrated that, in this solvent, *Pa*-MAP 1.9 was able to maintain its initial structure during the 100 ns, presenting only a few structural changes at the N-terminus (Fig. 7b). In comparison with the simulation in water, it was also possible to note decreased RMSD, RMSF, radius of gyration and SASA values, indicating improved structural stability in TFE (Fig. 6b). As expected, simulations in the presence of a micelle containing 128 SDS residues confirmed the preference of *Pa*-MAP 1.9 for amphipathic anionic environments, preserving its α-helical structure. In this simulation, as for 50% TFE, only a slight RMSD variation of 0.3 nm was observed, stabilizing then between 0.4 and 0.6 nm, indicating few deviations in the input and output structures along the 100 ns (Fig. 6c). Moreover, the radius of gyration was also equivalent to that for TFE simulation, remaining around 1.2 nm (Fig. 6c). In addition, SASA seemed to be conserved in these two simulations, varying from 19 to 23 nm during the 100 ns (Fig. 6c). However, contrary to what was obtained in 50% TFE, a more significant fluctuation could be observed at the C-terminus region of *Pa*-MAP 1.9 when in contact with SDS micelles, as demonstrated in Figs 6c and 7c.

**Figure 8:**
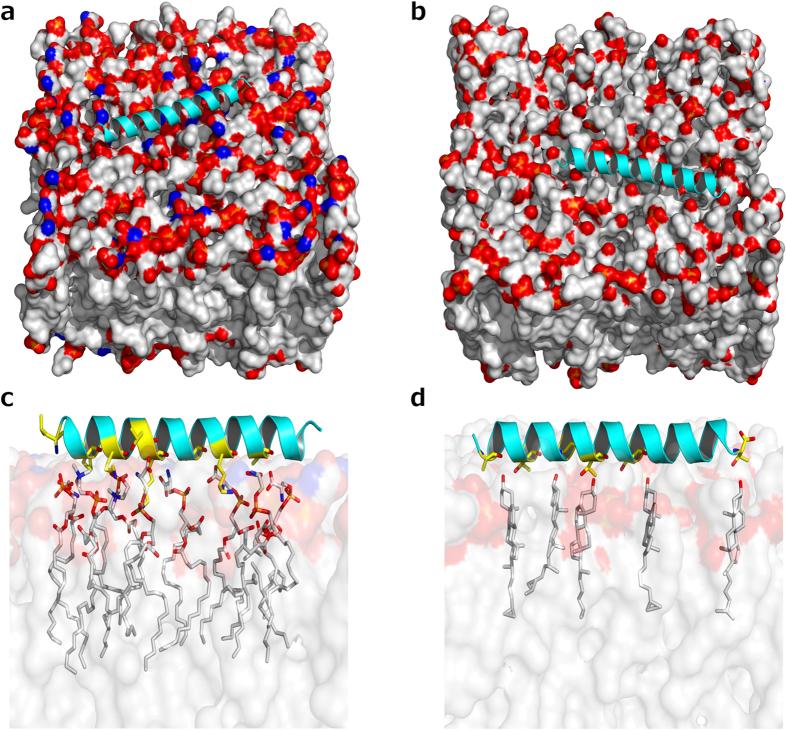
*In silico* interactions between *Pa*-MAP 1.9 and anionic/zwitterionic mimetic membranes. Three-dimensional theoretical representation of the complexes *Pa*-MAP 1.9–POPC/POPS (50:50) (**A**) and *Pa*-MAP 1.9–POPC/Chol (70:30) (**B**), as well as zoom images, revealing the amino acids residues from *Pa*-MAP 1.9 (yellow sticks) possibly involved in interactions with the phospholipids (white sticks) from both POPC/POPS (**C**) and POPC/Chol (**D**) mimetic membranes.

By using our best theoretical model for *Pa*-MAP 1.9, we also predicted the affinity and atomic interactions of this peptide with mimetic membranes containing the same lipid compositions experimentally tested on the leakage studies. Fifty runs of molecular docking were performed, and all peptide/membrane complexes were ranked by their affinity, in kcal.mol^−1^. The best affinity values for *Pa*-MAP 1.9 in POPC/POPS (Fig. 8a) and *Pa*-MAP 1.9 in POPC/Chol (Fig. 8b) were −5.4 and −3.7 kcal.mol^−1^, respectively, corroborating our experimental results, wherein *Pa*-MAP 1.9 seemed to interact better with anionic vesicles and Gram-negative bacteria with anionic lipid compositions. Furthermore, in both complexes, *Pa*-MAP 1.9 maintained a well-defined α-helical conformation during the simulations (Fig. 8a,b). In the complex of *Pa*-MAP 1.9 with POPC/POPS it was possible to predict 10 interactions (Fig. 8c) divided into hydrogen bonds (HB), involving nitrogen (N), oxygen (O/OG1) atoms of Leu1, Thr10, Lys11,18 and Ala15,22 from *Pa*-MAP 1.9, and saline bonds (SB), involving positively charged nitrogen atoms of the side chain (NZ) of Lys4,7,11,18 from *Pa*-MAP 1.9, ranging from 2.8 to 3.6 Å of distance (Table 2). On the other hand, in the complex of *Pa*-MAP 1.9 with POPC/Chol, only 6 HBs could be observed (Fig. 8d), involving O/OG1 atoms of Ala2 and Thr6,10,13,17,28 from *Pa*-MAP 1.9 and oxygen atoms (O3) from cholesterol, with distances between 2.7 and 3.4 (Table 2).

## Discussion

Here we describe the functional and structural characterization of *Pa*-MAP 1.9, a rationally-designed cationic AMP based on a multifunctional peptide analogue from *P. americanus*, denominated *Pa*-MAP^12^. In our antibacterial assays, *Pa*-MAP 1.9 revealed better activities mainly against Gram-negative bacterial strains (*E. faecalis*, *E. coli* and *K. pneumoniae*), with lower MIC against *E. coli* (6 μM) when compared with its precursor *Pa*-MAP (30 μM)^12^. Similar enhanced activities against Gram-negative strains have also been reported for other AMPs isolated or derived from the winter flounder *P. americanus*, as it is the case of pleurocidin, a cationic α-helical peptide with high activities against *E. coli* and *Pseudomonas aeruginosa*, with MIC values below 1 μM^16^. Moreover, other fish derived AMPs, such as pardaxin (*Purdachirus marmorutus*) and piscidins-1, -2, -3 and -4 (*Morone chrysops*; *Morone saxatilis*), were also active against the human pathogens *E. coli*, *Acinetobacter calcoaceticus*, *P. aeruginosa*, *S. typhimurium* and *Shigella flexneri*^18^,^20^.

In addition to direct antibacterial assays, we also studied the antimicrobial ability of *Pa*-MAP 1.9 in preventing biofilm growth, as well as combating pre-formed bacterial biofilms, since different physiological conditions occur in these consortiums when compared to planktonic bacteria, and this leads to increased antibiotic resistance. Previous studies have shown that anti-biofilm activity is independently determined compared to antibacterial activity against planktonic (free-swimming) bacteria. These assays revealed that *Pa*-MAP 1.9 is a promising anti-biofilm peptide with activity at concentrations 90-fold lower than the MIC against one strain of *Klebsiella pneumoniae*. Similar to our results, Tao and colleagues^15^ described the anti-biofilm potential of another *P. americanus* derived-peptide (pleurocidin) in combating *Streptococcus mutans* biofilm, causing a reduction in *S. mutans* biomass upon treatment with 11.8 μM of the peptide. In addition, a study performed by Choi and Lee^16^ reported that, below 0.7 μM, pleurocidin could inhibit pre-formed *E. coli* and *P. aeruginosa* biofilms, causing biomass reductions of 22.4 and 48.3%, respectively. Interestingly, the concentration of *Pa*-MAP 1.9 required to combat *E. coli* and *K. pneumoniae* biofilms were lower than its MIC values for the two studied strains. Similar findings were previously made for other small synthetic cationic peptides (IDR-1018, and DJK-6), in circumstances where MIC values against *K. pneumoniae* could not be determined (>64 μg.mL^−1^), but relevant anti-biofilm properties were detected at 2–4 μg.mL^−1^ (MBIC)^21^. Flow cell experiments showed even better results, with just 2 μg.mL^−1^ of IDR-1018 and DJK-6 being necessary to eliminate most pre-existing *K. pneumoniae* biofilms^21^. These findings were consistent with the concept that anti-biofilm peptides act by several mechanisms that include dispersing bacterial biofilms and triggering death of cells within biofilms, but that these activities involve independent mechanisms from those determining AMP activity.

When evaluated with regard to its potential for causing morphological damage to the surface of *E. coli*, *Pa*-MAP 1.9 showed dose-dependent action. Below its MIC, only slight changes were observed, suggesting that *Pa*-MAP 1.9 might insert into the *E. coli* cytoplasmic membrane and translocate to act on intracellular targets. However, substantial cell damage was observed upon 50-fold increasing its concentration suggesting lytic activity at concentrations above the MIC^22^. This behavior was also described for Sub3, an AMP optimized based on bovine bactenecin^23^ whereby it was found that Sub3 antimicrobial activity is intrinsically related to membrane binding and its cell-penetrating ability, since at 1 to 10 μM it did not affect the surface of *E. coli* strains^17^. Nevertheless, increasing the concentration of this peptide to 100 μM led to evident morphological damage, including bacterial surface disruption. However, as for *Pa*-MAP 1.9, such a high concentration was not required for the lethal activities of this peptide.

The results obtained in the AFM experiments encouraged us to study the ability of *Pa*-MAP 1.9 to interact with lipid vesicles mimicking different types of biological membranes. *Pa*-MAP 1.9 was effective in disrupting vesicles constituted with some proportion of anionic lipids such as POPS, POPG and LPS. However, as anticipated based on our bioassays, much higher concentrations were necessary for the disruption of vesicles composed solely of zwitterionic or neutral phospholipids (*e.g.*, POPC and cholesterol). Furthermore, cholesterol-containing membranes could not be significantly disrupted even at the highest concentrations tested. Apparently, cholesterol which is present in mammalian cells assists in the stability of these membranes^24^. *Pa*-MAP 1.9 lipid-selective disruption of biomembrane model systems was also consistent with our bioassays, wherein this peptide clearly presented better activities against Gram-negative bacteria, which contains a more anionic surface based on lipid composition. In addition, the observation that *Pa*-MAP 1.9 revealed no cytotoxicity against different mammalian cell lines could have been related in part to its inability to disrupt neutral (zwitterionic) phospholipids and cholesterol-containing membranes. Lee and colleagues^25^ observed similar results in studying detergent-like membrane disruption by four magainin analogues, namely MSI-78, MSI-367, MSI-594 and MSI-843. In this context, by using solid-state nuclear magnetic resonance (NMR), the authors concluded that MSI peptides fragmented LUVs by using a detergent-like process. As described here for *Pa*-MAP 1.9, MSI peptides were able to disrupt anionic (POPG; POPS) LUVs at much lower concentrations than zwitterionic (POPC) LUVs. However, when cholesterol was present in the lipid vesicle composition, the disruptive potential of these peptides drastically decreased.

Similarly, in molecular docking simulations, the complex of *Pa*-MAP 1.9 with POPC/POPS revealed improved affinity values and a larger number of atomic interactions of different types (hydrogen and saline bonds), when compared to the *Pa*-MAP 1.9 complex with POPC/Cholesterol (Table 2). In a recent work, the parent peptide *Pa*-MAP was predicted to form hydrophobic interactions with a DPPC membrane with a lower affinity value (−3.1 kcal.mol^−1^), when compared with the results here^22^. Comparing the primary sequences of *Pa*-MAP and *Pa*-MAP 1.9, it was possible to observe a decrease in the hydrophobic residue content of *Pa*-MAP 1.9 (64%) when compared to *Pa*-MAP (73%), which is due to the substitution of *Pa*-MAP hydrophobic residues by hydrophilic ones in *Pa*-MAP 1.9. Similarly this was reflected in the hydrophobic moment of *Pa*-MAP 1.9, which was higher when compared to *Pa*-MAP, being 0.26 and 0.10 on the Eisenberg scale, respectively^26^. Furthermore, due to the addition of four lysine residues (Lys4, Lys7, Lys11 and Lys18) 1.9 had a net charge of + 4, which made this peptide much more cationic than its precursor (net charge = −1), favouring its interaction with bacterial membranes. In addition, the positioning of the amino acid residues of *Pa*-MAP 1.9 was designed to allow a more efficient distribution of hydrophobic and positively charged faces, favoring the amphipathicity of the molecule, as well as distributing alanine residues more evenly along the entire peptide, increasing the probability of helical structuring. These characteristics of *Pa*-MAP 1.9 would provide a major driving force for α-helix insertion into anionic bilayers, causing their perturbation, as well as entropy loss due to peptide loss of water interactions caused by translocation into the membrane and consequent α-helix structuring, as observed previously for other polyalanine peptides^27^.

Structurally, the CD spectra of *Pa*-MAP 1.9 revealed a well-defined α-helix conformation in the presence of SDS or 50% TFE. Similarly, Perrin and colleagues^28^ described the CD spectrum of piscidins 1 and 3 which, in the presence of anionic vesicles (POPC/POPG 3:1 and POPE/POPG 1:1), tend to become arranged into α-helical structures. In water, however, Campagna and co-workers^19^ reported that piscidins 1 had an unstructured conformation, also observed by NMR spectroscopy. Consistent with this work, when *Pa*-MAP 1.9 was solubilized in water, independently of the pH, a random conformation could be observed. These events could also be three-dimensionally visualized in our molecular dynamics simulations, whereby *Pa*-MAP 1.9 presented a well-defined α-helix conformation when in 50% TFE and in contact with an SDS micelle, but a coil conformation with a small, central α-helix in water. These features are consistent both with other AMPs from polar fishes as with the polyalanine precursor *Pa*-MAP^12^. Such environment-dependent behavior is important not only for the understanding of membrane-based mechanisms of action, but also are crucial for the elucidation of how AMPs reorient, insert into and translocate across membranes to act on intracellular targets.

In summary, *Pa*-MAP 1.9 proved to be a useful candidate for the treatment of Gram negative bacteria infections, especially in their biofilm state. It works through a dose-dependent mechanism of action, without being hemolytic and cytotoxic against mammalian cells. We also demonstrated that *Pa*-MAP 1.9 was able to interact with bacterial mimetic membranes and vesicles, adopting a well-defined α-helical conformation that might favor *Pa*-MAP 1.9 orientation and insertion into lipid bilayers to perform its activities.

## Material and Methods

### *Pa*-MAP 1.9 synthesis and mass spectrometry analysis

*Pa*-MAP 1.9 was purchased from Peptide 2.0 Incorporated (USA), which synthesized the peptide at 95% purity (after TFA removal) by the stepwise solid-phase method using the N-9-fluorenylmethyloxycarbonyl (Fmoc) chemistry on a Rink amide resin. *Pa*-MAP 1.9 molecular mass was confirmed by MALDI-ToF MS/MS analysis (Autoflex, Bruker Daltonics, Billerica, MA). The synthetic peptide concentrations for all *in vitro* experiments were determined using the measurement of absorbance at 205, 215 and 225 nm.

### *In vitro* antimicrobial assays

For antimicrobial assays, strains of *E. coli* (ATCC 8739, KPC 001812446 and KPC 002101123), *K. pneumoniae* (KPC 001825971, KPC 002210477 and ATCC 13883), *E. faecalis* (ATCC 19433), *P. aeruginosa* (ATCC 27853) and *S. aureus* (ATCC 25923) were grown in Luria Bertani (LB) broth overnight, at 37°C. MIC measurements were performed in LB medium with 5 × 10^6^ CFU and dilutions of *Pa*-MAP 1.9 from 0.7 to 115 μM. MIC was assessed as the minimal 100% inhibitory concentration of peptide after 12 h of incubation at 37 °C. During this period, the absorbance was measured in a plate reader (Bio-Rad 680 Microplate Reader) at 595 nm every 30 min. Bacteria in LB medium and in several dilution of chloramphenicol were used as negative and positive controls, respectively. All experiments were performed in triplicate.

### Anti-biofilm assays

Dilutions (1/100) of overnight cultures of *E. coli* (ATCC O157) and *K. pneumoniae* (KPC 001825971) grown overnight in LB medium were incubated in basal medium 2 (BM2) [62 mM potassium phosphate buffer (pH 7), 7 mM (NH_4_)_2_SO_4_, 2 mM MgSO_4_, 10 μM FeSO_4_, 0.5% glucose], in 96-well cell culture cluster round bottom microplate in the presence of *Pa*-MAP 1.9 for 24 h at 37 °C. Positive growth control contained only bacteria, and negative control wells contained broth only. Afterwards, planktonic cells were removed and microplate wells were washed twice with deionized water. The attached bacteria remaining were stained with 100 μL of 0.1% (m/v) crystal violet for 20 min. The microplates was rinsed twice with deionized water, air-dried and resolubilized with 110 μL of ethanol 70%. The content of the microplate was transferred to a new microplate, and the MBIC of *Pa*-MAP 1.9 was accessed at 595 nm in a microtiter plate reader (Bio-Tek Instruments Inc., VT).

### Flow cell assays

Biofilms were grown for 72 h, at 37 °C in flow chambers with channel dimensions of 1 by 4 by 40 mm, as previously described^29^. For treatment of pre-formed biofilms, bacteria were allowed to develop structured 2-day-old biofilms prior to treatment with *Pa*-MAP 1.9, for the following 24 h. The medium used to allow biofilm growth was (BM2) containing 0.4% (wt/vol) glucose as a carbon source. Silicone tubing (VWR, 0.062 in ID by 0.125 in OD by 0.032 in wall) was autoclaved, and the system was assembled and sterilized by pumping a 0.5% hypochlorite solution through the system at 6 rpm for 30 min using a Watson Marlow 205S multi-channel peristaltic pump. The system was then rinsed at 6 rpm with sterile water and medium for 30 min each. Flow chambers were inoculated by injecting 400 μl of an overnight culture diluted at 1:25 (v/v). After inoculation, chambers were left without flow for 2 h, after which medium was pumped through the system at a constant rate of 0.5 rpm for 48 h. After this time, treatments were diluted in BM2 medium at desired concentration and pumped through the system for 24 h. Biofilm cells were stained using the LIVE/DEAD BacLight Bacterial Viability kit (Molecular Probes, Eugene, OR) prior to microscopy experiments. A ratio of SYTO-9 (green fluorescence, live cells) to propidium iodide (PI) (red fluorescence, dead cells) of 1:5 was used. Microscopy was done using a confocal laser-scanning microscope (Olympus, Fluoview FV1000) and three-dimensional reconstructions were generated using the Imaris software package (Bitplane AG).

### Hemolytic assay

The hemolytic activity of *Pa*-MAP 1.9 was assessed by using human erythrocytes approved by the ethics committee of FEPECS (number: 396.061) and registered in Brazil Platform (number: 16131213.0.0000.5553), according with the approved guidelines. The cells were stored at 4 °C. Erythrocytes were washed three times with 50 mM phosphate buffer, pH 7.4. The peptide solution was added to the erythrocyte suspension (1%, by volume), at a final concentration ranging from 0.7 to 115 mM in a final volume of 100 mL. Samples were incubated at room temperature for 60 min. Hemoglobin release was monitored by measuring the supernatant absorbance at 540 nm. Zero hemolysis control (blank) was determined with erythrocytes suspended in the presence of 50 mM phosphate buffer, pH 7.4, and for positive control (100% of erythrocyte lyses); an aqueous solution of 1% (by volume) triton X-100 dissolved in distilled water was used instead of the peptide solution. Hemolytic assays were performed in triplicate.

### Cytotoxicity assays

For the cytotoxic assays, confluent RAW 264.7 (mouse leukemic monocyte macrophage) cell line was challenged with 0.7 to 115 μM of *Pa*-MAP 1.9 and incubated at 37 °C in a 5% CO_2_ atmospheres for 48 h, according to previously described methodology^12^. Experiments were performed in triplicate and cells imaged using an inverted optical microscope (Leitz) in order to describe morphological alterations. The neutral red dye-uptake method^30^ was used to evaluate cell viability. Cells were incubated in the presence of 0.01% (by weight) neutral red solution for 3 h at 37 °C in a 5% CO_2_ atmosphere. After, the medium was removed and the cells were fixed with 4% formalin in phosphate-buffered saline, pH 7.2. The dye, incorporated by the viable cells, was eluted by using a mixture of methanol:acetic acid:water (50:1:49,v:v:v), and the dye uptake was determined by measuring the absorbance at 490 nm in an automatic spectrophotometer (ELx800 TM-Bio-TeK Instruments, Inc.). The cytotoxic assays were performed in triplicate.

### Bacteria preparation and atomic force microscopy imaging

The effects of *Pa*-MAP 1.9 on *E. coli* (ATCC 25922) were evaluated by AFM imaging as previously described^31^,^32^. Bacterial cells were incubated with *Pa*-MAP 1.9 at 0, 6 and 300 μM in growth broth, at 37 °C, for 1 h. A 100 μL droplet of each sample was applied onto poly-L-lysine-coated glass slide and left at 25 °C for 30 min. After deposition, the sample was rinsed 10 times with Milli-Q water, and left to air-dry. AFM images were obtained on a JPK Instruments NanoWizard II (Berlin, Germany) mounted on a Carl Zeiss Axiovert 200 inverted microscope (Jena, Germany). Images were performed in intermittent contact mode (air) using ACL silicon cantilevers from AppNano (Huntingdon, UK) with a tip radius of 6 nm, a resonant frequency of approximately 190 kHz and a spring constant of 58 N m^−1^. All images were obtained with similar AFM parameters (setpoint, scan rate and gain values). The scan rate was set between 0.3 and 0.6 Hz and the setpoint was close to 0.3 V. Height and error signals were collected and images were analyzed with the JPK image processing software v. 5.1.13.

### LUV preparation and leakage assays

Large unilamellar vesicles with ~100 nm of diameter were obtained by extrusion of multilamellar vesicles, as described elsewhere^33^. POPC, POPG and POPS were obtained from Avanti Polar Lipids (Alabaster, AL), while LPS (*E. coli* O26:B6) and cholesterol (Chol) were obtained from Sigma (St. Louis, MO). The LUVs studied were zwitterionic (pure POPC and POPC:Chol 70:30) or anionic (POPC:POPG 80:20, POPC:POPG 50:50, pure POPG, POPC:POPS 80:20, POPC:POPS 50:50, POPC:LPS 80:20 and POPC:POPG:LPS 50:30:20). For lipid film formation with LPS, it was dissolved in chloroform:methanol (2:1) and the solution was vortexed and bath sonicated at 40 °C for 15 min. Stock solutions were kept at 4 °C overnight before measurements. Phosphate buffer saline (20 mM sodium phosphate, 150 mM NaCl) pH 7.4 was used for these measurements.

Peptide-induced lipid vesicle leakage was measured as previously described^13^, by fluorescence spectroscopy, on a Varian Cary Eclipse fluorescence spectrophotometer (Mulgrave, AU). In this assay, we monitored the release of 5,(6)-carboxyfluorescein (CF; Sigma, St. Louis, MO), trapped in the LUV. LUVs were prepared as described above, with dried film hydration in phosphate buffered saline (PBS) containing 100 mM CF (pH was adjusted to 7.4 with NaOH). Free CF was removed by passing the suspension through an EconoPac 10 DG column from Bio-Rad (Richmond, CA), where the vesicles are eluted with the void volume^13^. Aliquots of the liposomal stock preparations (diluted to 10 μM) were incubated with different concentrations of the peptide at 25 °C. Fluorescence was recorded continuously for 30 min, with excitation at 492 nm and emission at 517 nm (5 and 10 nm excitation and emission slits, respectively).

### Circular dichroism

CD measurements were performed on a JASCO J-815 spectropolarimeter (Easton, MD), equipped with a Peltier-type temperature controller and a thermostable cell holder, also interfaced with a thermostatic bath. Spectra were recorded in 0.1 cm path length quartz cells at a peptide concentration ranging from 0.05 to 0.5 mg.mL^−1^ in 2 mM Na-acetate buffer at pH 3.0, 2 mM Na-acetate buffer at pH 4.0, 2 mM Na-acetate buffer at pH 5.5, deionized water (Milli-Q), 2 mM Tris-HCl buffer at pH 7.0, 2 mM Tris-HCl buffer at pH 8.5, 2 mM glycine-NaOH buffer at pH 10.0 and 2 mM glycine-NaOH buffer at pH 11.0. Four consecutive scans were accumulated and the average spectra stored. *Pa-*MAP 1.9 analysis in the presence of SDS and TFE were performed in the same quartz cell with a 0.1 cm path length at 20 °C. The spectra were recorded between 190 and 260 nm at a scan speed of 50 nm.min^−1^ and four scans were performed per sample. The spectra were recorded in three average environments: distilled water, 28 mM SDS and TFE 50% in water (v:v). The observed ellipticity was converted into the mean residue ellipticity [θ] based on a mean molecular mass per residue of 115 Da. The data were corrected for the baseline contribution of the buffer and the observed ellipticities at 222 nm were recorded. The α-helical content of the peptides was calculated from mean residual ellipticity at 222 nm ([θ]_222_) using the following equation: f_H_ = [θ]_222_/[−40,000(1 − 2.5/n)], where f_H_ and n represent the α-helical content and the number of peptide bonds, respectively^34^. All spectra were acquired at 37 °C.

### Molecular Modelling

Initially, BLASTp^35^ was performed in order to find the best primary sequence template for molecular modelling. After that, 200 theoretical three-dimensional models were constructed by using Modeller v. 9.12^36^, based on an antifreeze peptide primary sequence (PDB code: 1WFA) isolated from *P. americanus.* The lowest free-energy theoretical model for *Pa*-MAP 1.9 was then selected and used for validation procedures. Firstly, PROCHECK^14^ evaluated peptide’s geometry, stereochemistry and energy distribution, also calculating its average score for dihedral angles, jointly with covalent forces. Moreover, 3DSS^37^ was also used for (RMSD) calculation by superimposing Cα-traces and backbones of both theoretical and template tridimensional structures.

### Molecular Dynamics

The molecular dynamics simulations for the *Pa*-MAP1.9 were carried out in three different steps. The first one was performed in water; the second one in water and TFE 50%/50% (volume/volume) and, the third one was carried out in water with an anionic environment (SDS micelle). All simulations were performed by using the computational package GROMACS v.4^38^ with the GROMOS96 43A1 force field implemented. As initial structure for dynamics simulations the best tridimensional theoretical model of *Pa*-MAP 1.9 was used, which was immersed in 7,897 water molecules in a cube box with sides measuring 6.22 nm. In order to neutralize the system’s charge, chloride ions were also added (four ions in this case). TFE 50% simulation was carried out with 1,892 and 2,023 water molecules, as well as 459 and 354 TFE molecules in a cube box with sides measuring 4.78 nm. The final step (SDS) was carried out in a dodecahedral box with a minimum distance of 0.7 nm between the non-water molecules and the box's frontiers. In this case, *Pa*-MAP 1.9, a SDS micelle with 128 phospholipid residues, 13,021 water molecules and chlorine ions constituted the system. Geometry of water and water/TFE 50% molecules was constrained using the SETTLE algorithm^39^. Moreover, the LINCS algorithm was used to link all the atom bond length. Particle Mesh Ewald (PME) was also used for electrostatic corrections, with a radius cut-off of 1.4 nm in order to minimize the computational simulation time. The same radius cut off was also used for van der Waals interactions. The list of neighbors of each atom was updated every 10 simulation steps of 2 fs each. A conjugate gradient (2 ns) and the steepest descent algorithms (2 ns) were implemented for energy minimization. After that, the system underwent a normalization of pressure and temperature, using the integrator stochastic dynamics (SD), 2 ns each. The system with minimized energy and balanced temperature and pressure was carried out using a step of position restraint, using the integrator Molecular Dynamics (MD), for 2 ns. The simulations were carried out at 27 °C *in silico*, aiming to understand the structural conformation of the peptide more nearly to that observed *in vitro* bioassays. The total time of *Pa*-MAP 1.9 simulation was 100 ns, in triplicate.

### Molecular Docking

For molecular docking studies, the program AUTODOCK 4.2^40^ was used in order to predict the possible modes of interaction between *Pa*-MAP 1.9 and anionic as well as zwitterionic mimetic membranes, based on what was done in the leakage experiments. For this, the CHARMM-GUI^41^ server was used for membrane construction, constituted of POPC/POPS (50:50) (anionic environment) and POPC/cholesterol (70:30) (zwitterionic environment), with 75 × 75 × 45 Å^3^ of dimension. Moreover, by using AutoDock Tools, all the hydrogen atoms were added and a grid box of 60 × 60 × 20 points with spacing of 1.0 Å was centered on the membrane surfaces. Furthermore, AutoDock Tools were also used for peptide manipulation, where the maximum freedom to side chains was unlocked. The molecular docking simulations were programed for fifty random runs, with the generated structures being ranked based on their affinity values in kcal.mol^−1^. After all simulations, PyMOL was used to predict peptide-membrane interactions, respecting the distance of 3.6 Å for all atoms involved.

## Additional Information

**How to cite this article**: Cardoso, M. H. *et al.* A polyalanine peptide derived from polar fish with anti-infectious activities. *Sci. Rep.* **6**, 21385; doi: 10.1038/srep21385 (2016).

## Supplementary Material

Supplementary figure S1

## Figures and Tables

**Table 1:** Antibacterial, anti-biofilm, cytotoxic and hemolytic activities of *Pa*-MAP 1.9.

Bacterial strains	MIC for *Pa*-MAP 1.9 (μM)
*Enterococcus faecalis* (ATCC 19433)	1.5
*Escherichia coli* (ATCC 8739)	6
*Escherichia coli* (KPC 001812446)	6
*Escherichia coli* (KPC 002101123)	12
*Klebsiella pneumoniae* (ATCC 13883)	24
*Klebsiella pneumoniae* (KPC 002210477)	24
*Klebsiella pneumoniae* (KPC 001825971)	96
*Pseudomonas aeruginosa* (ATCC 27853)	>115
*Staphylococcus aureus* (ATCC 25923)	>115
**Bacterial strains**	**MBIC (μM)**
*Escherichia coli* (ATCC O157)	3
*Klebsiella pneumoniae* (KPC 001825971)	1.1
**Cell line**	**Cytotoxic activity (μM)**
RAW 264.7 (mouse leukemic monocyte macrophage)	>115
**Cell type**	**Hemolytic activity (μM)**
Human erythrocytes	>115

**Table 2:** *In silico* predicted interactions between *Pa*-MAP 1.9 and anionic/zwitterionic mimetic membranes revealing the types and distances of atomic interactions occurring in these complexes.

*Pa*-MAP 1.9			Distances (Å)	POPC/POPS (50:50)			Interactions
Residues	Positions	Atom Names		Lipids	Positions	Atom Names	
Leu	1	N	3.6	POPS	27	O14	HB
Lys	4	NZ	3.5	POPC	10	O14	SB
Lys	7	NZ	3.6	POPC	18	O13	SB
Thr	10	OG1	3.1	POPS	6	O13	HB
Lys	11	NZ	3.6	POPS	6	O13	SB
Lys	11	O	3.3	POPS	3	N	HB
Ala	15	O	3.5	POPS	2	O13	HB
Lys	18	NZ	3.5	POPS	4	O13	SB
Lys	18	O	3.5	POPS	4	O13	HB
Ala	22	O	2.8	POPS	9	O13	HB
***Pa*****-MAP 1.9**				**POPC/Chol (70:30)**			
Ala	2	O	2.7	Chol	7	O3	HB
Thr	6	OG1	3.0	Chol	7	O3	HB
Thr	10	OG1	2.8	Chol	2	O3	HB
Thr	13	OG1	3.4	Chol	9	O3	HB
Thr	17	O	3.2	Chol	15	O3	HB
Thr	28	OG1	2.7	Chol	50	O3	HB
