# Corrigendum: A polyalanine peptide derived from polar fish with anti-infectious activities

**DOI:** 10.1038/srep28216

**Published:** 2016-07-20

**Authors:** Marlon H. Cardoso, Suzana M. Ribeiro, Diego O. Nolasco, César de la Fuente-Núñez, Mário R. Felício, Sónia Gonçalves, Carolina O. Matos, Luciano M. Liao, Nuno C. Santos, Robert E. W. Hancock, Octávio L. Franco, Ludovico Migliolo

Scientific Reports 6: Article number: 2138510.1038/srep21385; published online: 02 26 2016; updated: 07 20 2016

In this Article, there is a typographical error in the Results section.

“*Pa*-MAP 1.9 (NH2-LAAKLTKAATKLTAALTKLAAALT-COOH) was designed …”

should read:

“*Pa*-MAP 1.9 (NH2-LAAKLTKAATKLTAALTKLAAALTAAAT-COOH) was designed …”

