# Supplementary material for: A polyalanine peptide derived from polar fish with anti-infectious activities
Source: Sci Rep. 2016 Feb 26;6:21385. doi: 10.1038/srep21385 (PMC4768251; doi:10.1038/srep21385)

Supplementary Information

A polyalanine peptide derived from polar fish with anti-infectious activities

Marlon H. Cardoso, Suzana M. Ribeiro, Diego O. Nolasco, César de la Fuente-Núñez, Mário R. Felício, Sónia Gonçalves, Carolina O. Matos, Luciano M. Liao, Nuno C. Santos, Robert E. W. Hancock, Octávio L. Franco and Ludovico Migliolo

Supplementary figure:

Figure S1. **Mass spectrometry analysis of *Pa*-MAP 1.9**. Ion of monoisotopic mass $[M+H]^+$ of 2668.0 m/z is represented.

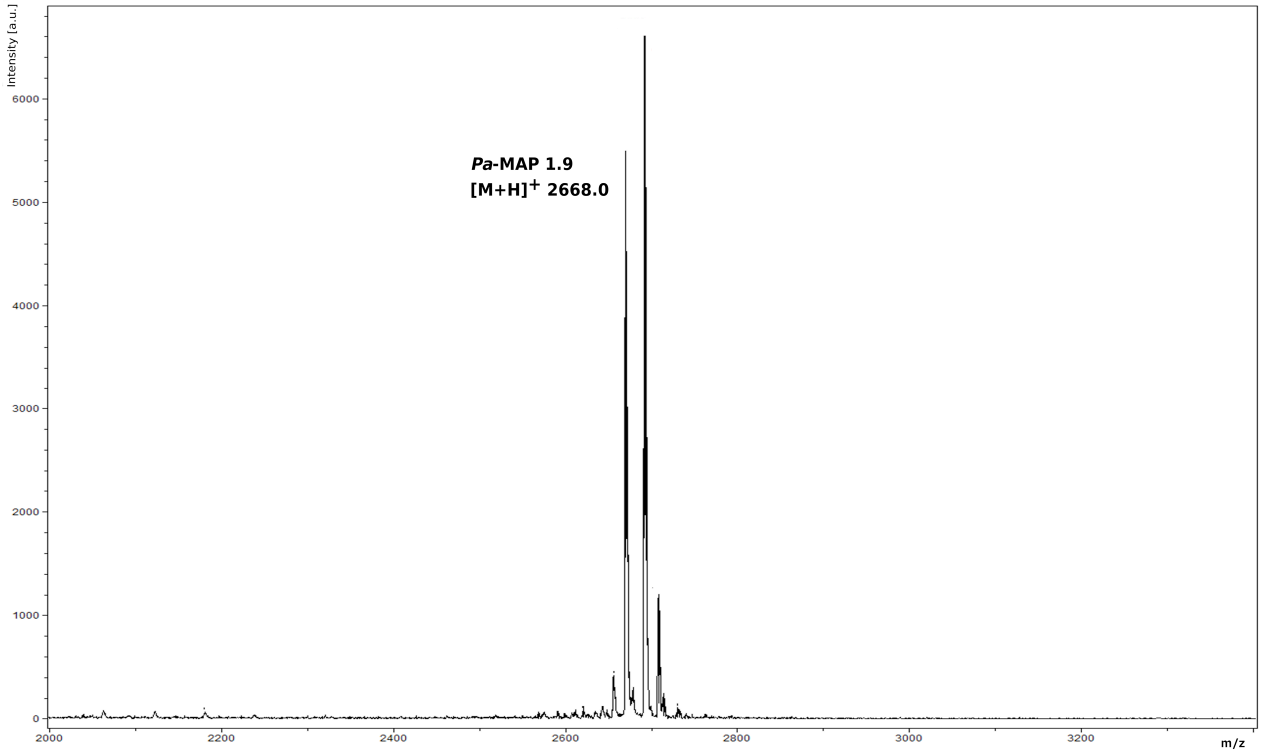

Supplement: Supplementary figure S1 [file srep21385-s1.pdf]
